# Limbic Encephalitis With Dual Positivity

**DOI:** 10.7759/cureus.40399

**Published:** 2023-06-14

**Authors:** Esra Erdil Yucesoy, Handenur Tunc, Sema Nur Erdem, Suheyla Bozkurt, Nese Tuncer

**Affiliations:** 1 Neurosciences, King's College Hospital, London, GBR; 2 Department of Neurology, Marmara University Pendik Training and Research Hospital, İstanbul, TUR; 3 Department of Neurology, Marmara University Pendik Training and Research Hospital, Istanbul, TUR; 4 Department of Pathology, Marmara University Pendik Training and Research Hospital, Istanbul, TUR

**Keywords:** onco-immunology, lung cancer, onconeural antibodies, paraneoplastic neurological syndromes, autoimmune limbic encephalitis

## Abstract

Limbic encephalitis is a well-defined clinical disorder among paraneoplastic neurological syndromes. Although it is not always possible to identify specific autoantibodies in limbic encephalitis, the presence of anti-neuronal nuclear antibody type 1 (ANNA1 or anti-Hu), anti-Ma2, collapsin response mediator protein 5 (CRMP-5-IgG or anti-CV2), anti-GABA_B_ receptors and anti-amphiphysin antibodies are often detected.

A 66-year-old male patient with complaints of forgetfulness was evaluated in our clinic after having seizures. In the neurological examination, the patient was found to be confused. In cranial MR fluid-attenuated inversion recovery (FLAIR) and T2-weighted images, the right hippocampal and parahippocampal structures showed hyperintense areas complying with limbic encephalitis. He had improvement with a course of 2 g/kg intravenous immunoglobulin (IVIG) followed by high-dose methylprednisolone therapy. Following the high-dose methylprednisolone therapy, anti-PCA1 (Yo) and anti-amphiphysin antibodies were positive and the tissue pathology report confirmed combined small-cell carcinoma and large-cell neuroendocrine carcinoma of the lung. In recent years, paraneoplastic neurological syndromes are better recognized with the identification of specific antibodies and the ubiquitous information on pathogenesis. This is the first known report in the literature that a case with both positive anti-PCA1 (Yo) and anti-amphiphysin antibodies together and underlying small-cell and large-cell neuroendocrine carcinomas.

## Introduction

Paraneoplastic encephalitis is a relatively common and well-defined disorder. Limbic or brainstem encephalitis is the usual clinical presentation, which may manifest as memory impairment, behavioural disturbance, mood disorder, and seizure. Acute or subacute onset of neuropsychiatric symptoms should be considered warning symptoms.

Short-term memory impairment typically occurs early during the course of the disease. The main differential diagnoses for limbic encephalitis include, but are not limited to, vasculitic diseases such as systemic lupus erythematosus, viral encephalitis, cerebrovascular diseases, psychiatric disorders, and metabolic encephalopathy. Certain laboratory findings, such as cerebrospinal fluid (CSF) testing, electroencephalographic (EEG) evaluation, cranial MRI findings, and detection of previously identified antibodies, are potential clues for the correct diagnosis of limbic encephalitis.

Although it is not always possible to detect specific autoantibodies in paraneoplastic limbic encephalitis, a variety of antibodies such as anti-neuronal nuclear antibody type 1 (ANNA1 or anti-Hu), anti-Ma2, collapsin response mediator protein 5 (CRMP-5-IgG or anti-CV2), anti-voltage-gated potassium channel antibody (VGKC), and anti-amphiphysin antibodies can be seen [1]. Anti-PCA1 (Yo) is typically associated with other types of paraneoplastic neurological syndromes such as paraneoplastic cerebellar degeneration. While the most common concurrent malignancy is small-cell lung cancer (SCLC), other malignancies, such as seminoma, thymoma, breast cancer, and lymphoma, can also be seen [2]. This article presents a case of anti-PCA1 and anti-amphiphysin antibody coexistence along with small-cell carcinoma and large-cell neuroendocrine carcinoma, which has not been previously reported in the literature.

## Case presentation

A 66-year-old male patient presented to our clinic with introversion and amnesia that started about eight months ago after having seizures. Three months ago, he presented to another clinic with amnesia, received a pre-diagnosis of limbic encephalitis and was treated with 2 g/kg intravenous immunoglobulin (IVIG). According to the information provided by his relative, his complaints decreased slightly after discharge but worsened again in the following weeks. The patient had known diagnoses of diabetes mellitus, hypertension, and atrial fibrillation.

**Figure 1:**
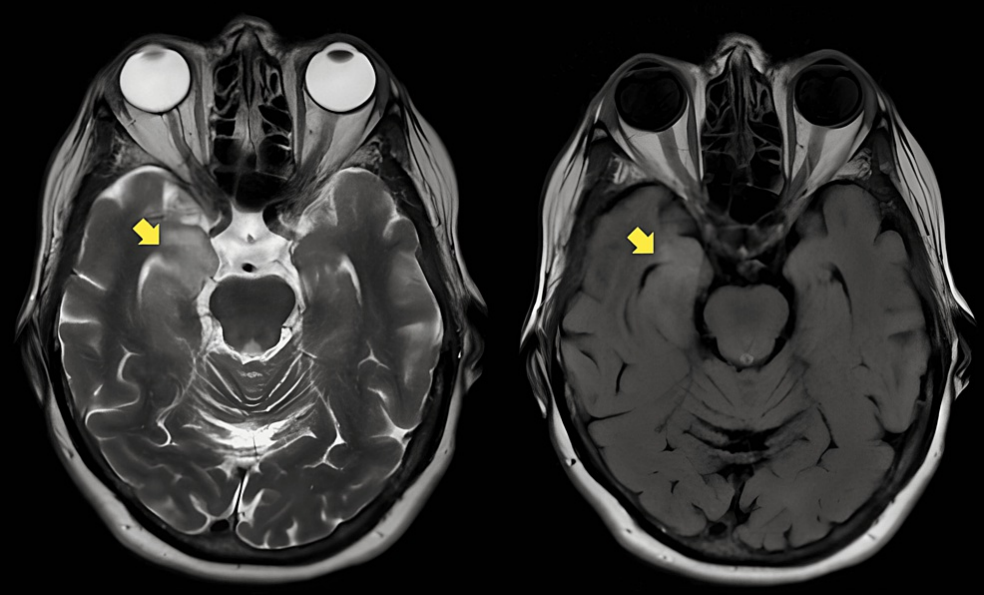
Cranial T2 and T2 FLAIR MR images showing hyperintensity in the right hippocampus and parahippocampus (yellow arrow) FLAIR: fluid-attenuated inversion recovery

His neurological examination revealed confusion, with the disorientation of place and time without any focal neurologic deficit. He scored 11/30 on the standardized mini-mental test (SMMT) (low performance in orientation, delayed recall, language, and constructional praxis sections). The cranial magnetic resonance images showed T2 fluid-attenuated inversion recovery (FLAIR) and T2 hyperintense areas without contrast enhancement in the right hippocampal and parahippocampal regions (Figure 1). The interictal EEG showed significant epileptiform discharges in the right centroparietal region (C4-P4). He was treated with levetiracetam at a dose of 3000 mg/day as antiseizure monotherapy. The cerebrospinal fluid findings were as follows: protein: 46.5 mg/dL (normal range: 15-45 mg/dL); glucose: 56 mg/dL; cell count: 24/mm³ mononuclear with 2/mm³ polymorphonuclear leukocytes. Infection parameters, including CSF and blood cultures, were negative.

The patient was diagnosed with autoimmune encephalitis since his clinical presentation, imaging findings, and CSF and EEG results could not be better explained by another neurological disease or other medical condition. The patient who achieved partial improvement with 2 g/kg IVIG treatment was treated with 500 mg/day pulse methylprednisolone therapy for three days after two weeks. Subsequently, he was initiated on 1 mg/kg oral methylprednisolone maintenance treatment.

**Figure 2:**
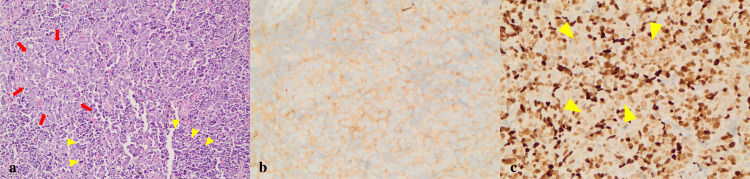
a. Nested large tumoural cells (red arrows) and small cells placed peripheral to large cells (yellow arrowhead) (hematoxylin and eosin, × 200 magnification). b. Diffuse cytoplasmic synaptophysin immunoreactivity in both tumour cell types (synaptophysin, × 400 magnification). c.Diffuse nuclear TTF-1 immunoreactivity in both tumour cell types (TTF-1, × 400 magnification)

The patient underwent positron emission tomography (PET)-CT for malignancy screening. Upon the detection of multiple suspicious areas with high fluorodeoxyglucose (FDG) uptake in the mediastinum, a bronchoscopy was performed for a lymph node biopsy. Histological evaluation of the lymph node biopsy showed neoplastic infiltration composed of two types of tumour cells. One of them was characterized by a nested growth pattern of large cells with eosinophilic cytoplasm, round to oval nuclei with vesicular chromatin and distinct nucleoli. The other type of neoplastic proliferation was composed of small cells with scant cytoplasm and oval nuclei with densely hyperchromatic chromatin. Nuclear moulding was characteristically seen among the small cells (Figure 2a). Small cells were placed around the nested large cells. Immunohistochemically, both types of tumour cells demonstrated cytoplasmic pan-cytokeratin, synaptophysin, and nuclear TTF-1 immunopositivity (Figures 2b, 2c). Diffuse cytoplasmic synaptophysin immunoreactivity in both types of tumour cells confirmed neuroendocrine differentiation in both large and small cell components of neoplastic infiltration.

The paraneoplastic syndrome panel screening performed by the immunofluorescence assay/line blot method showed anti-PCA1 and anti-amphiphysin positivity in the patient’s serum. The anti-PCA1 antibody was positive with a density of 35 (++, 1/100) and the anti-amphiphysin antibody was strongly positive with a density of 59 (+++, 1/1000). Other antibodies were negative (Table 1). After the administration of IVIG and subsequent methylprednisolone therapy, the patient clinically achieved significant neurological improvement. His SMMT score increased to 24/30 (low orientation and recall performance). Right after IV pulse methylprednisolone treatment, the patient was started on chemotherapy and radiotherapy for malignancy and is still on chemotherapy and antiseizure therapy.

**Table 1:** Autoimmune encephalitis panel results

Antibody	Result - Density
Anti-PCA1	Positive - 35 (++, 1/100)
Anti-Amphiphysin	Positive - 59 (+++, 1/1000)
Anti-CV1	Negative
Anti-PNMA2 (Ma2/Ta)	Negative
Anti-Ri	Negative
Anti-VGKC	Negative
Anti-NMDA	Negative
Anti-AMPAR1	Negative
Anti-AMPAR2	Negative

## Discussion

Paraneoplastic neurological syndromes have recently been better recognized with the identification of specific antibodies and the ubiquitous information on pathogenesis. Although no certain antibody clearly points to a specific malignancy or syndrome, it can guide the diagnosis of paraneoplastic encephalitis and malignancy screening for further investigation and follow-up. In addition, it is very important to follow up on limbic encephalitis patients with repeated screenings for malignancy in case of negative initial screening.

Neuronal autoantibodies target intracellular antigens and include an antineuronal nuclear antibody, type 1 (ANNA1 or anti-Hu) antineuronal nuclear antibody, type 2 (ANNA2 or anti-Ri) antineuronal nuclear Ab, type 3 (ANNA3) antiglial nuclear antibody, Purkinje cell cytoplasmic antibody, type 1 (PCA1 or anti-Yo), Purkinje cell cytoplasmic antibody, type 2 (PCA2), Purkinje cell cytoplasmic antibody, type Tr (PCATR), amphiphysin Ab, and collapsin response mediator protein 5 (CRMP-5-IgG or anti-CV2). These antibodies usually lead to a T-cell-mediated immune response [3].

Amphiphysin is an intracellular synaptic vesicle protein discovered in 1992 [4]. It was described in patients with paraneoplastic stiff-person syndrome in 1993 [5]. While this antibody was first attributed to the stiff-person syndrome, several case reports and small case series have later demonstrated that the anti-amphiphysin antibody is associated with limbic encephalitis, brainstem encephalitis, peripheral neuropathy, myelopathy, and underlying breast cancer and ovarian malignancies, and small cell lung cancer [6-8]. This led to the emergence of the term ‘non-stiff anti-amphiphysin syndrome’. This antibody is among those that target intracellular synaptic proteins, and these antigens are believed to cause an antibody immune response leading to impaired synaptic vesicle fusion and re-uptake.

Among the antibodies detected to be positive, anti-PCA1 is typically associated with paraneoplastic cerebellar degeneration and targets the cytoplasmic components of cerebellar Purkinje cells. The majority of cases have been reported in women with breast, ovarian, and Mullerian duct malignancies, different from our patients'. Anti-PCA1 is the most common antibody in paraneoplastic cerebellar degeneration. However, it can be related to many other paraneoplastic neurological syndromes, including limbic encephalitis.

The unusual findings in this limbic encephalitis case are the histologic coexistence of two different malignancies and the presence of two different paraneoplastic antibodies. Small-cell and large-cell carcinomas are among the high-grade carcinomas [9]. About 10-20% of large cell neuroendocrine carcinomas are commonly seen in combination with other lung tumours, more frequently with adenocarcinomas. In addition, the similar gene expression profiles of large-cell neuroendocrine carcinoma and SCLC have suggested that SCLC can be derived from large-cell neuroendocrine carcinoma [10].

There are overlapping syndromes between different neuronal autoantibodies; therefore, it is more practical to use a comprehensive panel for screening [11]. The growing number of recognized autoimmune/paraneoplastic antibodies and associated syndromes had led to a dramatic increase in the frequency of testing, resulting in an increase in the number of seropositive cases. There is a lack of information about the percentage of the presence of each antibody in limbic encephalitis in the literature. As in our case, a clinically less relevant autoantibody can be detected, making the results difficult to interpret. In fact, a recently published study elucidated the importance of multiple antibodies that are associated with low clinical relevance [11]. In our case, additional immunostaining to show the antigenic similarity to PCA-1 or amphiphysin was not performed on the pathological tissue. This further investigation would be a great contribution to future studies.

Serum and CSF samples should be analysed for paraneoplastic antibodies, and if the clinical presentation is inconsistent with the detected antibody, the antibody test should be repeated for the possibility of false positivity. In our case, the presence of antibodies was not checked twice; however, the first test revealed strong positivity. In limbic encephalitis cases, it is of great importance to repeat the screening for malignancy with a strict follow-up, when the initial screening is negative. It should be kept in mind that the presence of antibodies against the same antigens can be detected in different clinical conditions and that already-defined clinical syndromes may be associated with other antibodies that are not yet known.

## Conclusions

Patients with malignancy may present with neurological symptoms. In suspected cases, screening for malignancy and a strict follow-up if the first screening results are negative are of great importance. Immunotherapies are only available if the cases are recognised. Thus paraneoplastic neurological syndromes are accepted as not-to-miss conditions. Comprehensive antibody panels are more likely to capture causative antibodies. Even though some already-defined neurological syndromes may be associated with other antibodies that are not yet known, paraneoplastic neurological syndromes can have dual antibody positivity as presented in this case.
